# Two new species of gorgonian octocorals from the Tropical Eastern Pacific Biogeographic Region (Cnidaria, Anthozoa, Gorgoniidae)

**DOI:** 10.3897/zookeys.350.6117

**Published:** 2013-11-14

**Authors:** Odalisca Breedy, Gary C Williams, Hector M Guzman

**Affiliations:** 1 Centro de Investigación en Ciencias del Mar y Limnología, Universidad de Costa Rica. Centro de Investigación en Estructuras Microscópicas, P.O. Box 11501-2060, Universidad de Costa Rica, San José, Costa Rica; 2 Department of Invertebrate Zoology and Geology, California Academy of Sciences, 55 Music Concourse Drive, San Francisco, California, 94118, USA; 3 Smithsonian Tropical Research Institute, P.O. Box 0843-03092, Panama, Republic of Panama

**Keywords:** *Eugorgia*, eastern Pacific, gorgonian, soft corals, taxonomy, white species

## Abstract

The gorgoniid *Eugorgia* is exclusively an eastern Pacific genus. It has a wide geographic and bathymetric range of distribution, found from California to Perú and extends down to 65 m deep. Two new species are herein described. The morphological characters were analyzed and illustrated by light and scanning electron microscopy. *Eugorgia beebei* **sp. n.** can be distinguished by its white, ascending, sparse colony growth. *Eugorgia mutabilis* **sp. n.** can be distinguished by its white colony that changes color after collection, and the conspicuous sharp-crested disc sclerites. From a morphological point of view the new species are related to the *daniana*-group, the *rubens*-group and the *siedenburgae*-group of Eugorgia; their affiliations, and the proposal of a new group are discussed. These new species increases the number of species in the genus to 15, and contribute to the knowledge of the eastern Pacific octocoral biodiversity.

## Introduction

*Eugorgia* is a gorgonian octocoral (family Gorgoniidae) with 13 valid species. The genus is considered to be exclusively eastern Pacific and is distributed from southern California to Perú, and found in oceanic islands. It presents a wide bathymetric range of distribution, found in shallow waters (down to 40 m), and in the mesophotic region (down to 65 m) (Breedy and Guzman 2013). *Eugorgia* is characterized by having flabellate to bushy colonies with one or multiple planes. Branching is lateral, partially dichotomous, or pinnate-like, often bushy, and combinations of them; branch anastomosis is absent, but pseudo-anastomosis frequently occurs (Breedy et al. 2009). Colonies could be attached to hard substrates, debris, and coarse sand or muddy sediments. Polyps are fully retractile into the coenenchyme in slightly raised to prominent mounds arranged in series of longitudinal rows, or evenly distributed on the branches. Coenenchymal sclerites are of various types: spindle, disc-spindle, capstan, and the most dominant form that defines the genus is the characteristic double disc. Double discs could be incomplete, when the sclerite tubercles have a partial fusion, or complete, when the fused tubercles of the sclerites form wheels like flying saucers. Anthocodial sclerites are rarely found. The color of the sclerites is variable: brownish, orange, red, violet, white, yellow or combinations of these (Breedy et al. 2009). The colonies are orange, pink, purple, red, white, or yellow, some have with colored rings around the polyp mounds. They are produced by the arrangement of darker or lighter color sclerites around the polyp aperture, in some cases they are not surrounding the polyps, just sparsely distributed giving a sprinkled appearance to the branches. According to the morphological features, the species are proposed to form three groups, the *daniana*-group, the *ampla*-group and the monospecific *rubens*-group (see Breedy et al. 2009). A new group characterized by bushy, irregularly pinnate, bicolored colonies has been proposed for the recently described species *Eugorgia siedenburgae* Breedy & Guzman, 2013.

*Eugorgia* is recognised for their bright colored colonies. The white color has been reported only for one species, *Eugorgia alba* Bielschowsky, 1929 in the *ampla*-group (Breedy et al. 2009), although white specimens have been observed either in collections or in the field. Herein we describe two new species that were previously recorded as color varieties (Breedy et al. 2009, E. Deichmann as a museum label).

## Materials and methods

### Repository abbreviations

CAS California Academy of Science, California, USA

UCR Museo de Zoología, Universidad de Costa Rica

STRI Smithsonian Tropical Research Institute, Panama

USNM National Museum of Natural History, Washington, USA

The specimens used in this study belong to the octocoral collections of the above cited museums.

### Morphological analysis

Preserved specimens were photographed for later detailed observation. Sclerites were obtained by dissolving tissues from branches with 3.5% sodium hypochlorite (household bleach). Sclerites were rinsed many times with distilled water then 100% ethanol, dried, and mounted on stubs for scanning electron microscopy (SEM), and coated with 60–80 nm Pt/Pd. They were observed and photographed using an Hitachi 3700 SEM operated at 15kV. For light microscopy, clean sclerites were mounted in water or glycerin and observed and photographed using an Olympus LX 51 inverted stereoscope.

We followed Verrill (1868) and Breedy et al. (2009) for characters assessment. The terminology used in descriptions mostly follows Bayer et al. (1983), Breedy and Guzman (2002), and Breedy et al. (2009).

Morphological characters of colonies and the most abundant sclerite types of the species examined here are presented in Table 1. The most abundant sclerites in these species are disc-spindles and double discs that present various degrees of tubercle fusion. The illustrations of the sclerites are presented in different planes to provide a better idea of their architecture (Figs 3, 5). Comparison is made with the closest morphological groups, in this case, the *daniana*-, *siedenburgae*-, and *rubens*-groups (Table 1).

**Table 1.** Comparative features of the new species, *Eugorgia beebei* sp. n. and *Eugorgia mutabilis* sp. n. within the *daniana-* group, the *rubens*-group, and the *siedenburgae*-group. Characteristics are based on the holotypes and lectotypes (Verrill 1868, Breedy et al. 2009, Breedy and Guzman 2013). Sclerite sizes represent the maximum length or the common range found in the samples. Measurements are given in mm.

Species	Colony growth	Branching type	Max number. of branching	Pinna-like arr branchlets	Branchlet distance	Branchlet diameter	Branchlet length	Polyp distribution	Double disc	Capstans	Disc-spindle	Spindles	Bent spindles	Crosses	Anth. rods	Colour of colony	Bicolour colony	Coenenchymal sclerite colour	Colour rings
*Eugorgia aurantiaca* (Horn, 1860)	fla	irr-pi	6	X	1.5–8	1–2.5	6–30	irr	0.04–0.07	0.07	no	0.11	X	0.06 × 0.06	not found	do, r		r, y	y
*Eugorgia daniana* Verrill, 1868	fla	irr-pi	7	X	1–4	1–1.5	1–15	irr	0.065–0.08	0.07	0.13	0.13	X	0.075 × 0.065	not found	r		r, y	
*Eugorgia multifida* Verrill, 1870	fla	irr-pi	7	X	1–4	1–1.5	1–10	reg	0.07	0.07	0.13	0.13	X	0.06 × 0.06	0.08 mm	do, r		r, y	
*Eugorgia rubens* Verrill, 1868	spa	lb	5	X	6–20	1.5–2	2–30	reg	0.06–0.07	no	0.10	0.10		no	not found	p		p	
*Eugorgia siedenburgae* Breedy & Guzman, 2013	bu	irr-pi	10	no	1–15	1–1.5	2–30	irr	0.08–0.05	0.07	0.11	0.11		0.08 × 0.07	not found	p,o	X	p,y	
*Eugorgia mutabilis* sp. n.	fla	irr-pi	7	X	1–4	1–2	1–10	irr	0.045–0.075	no	0.15	0.14		no	not found	w		w	
*Eugorgia beebei* sp. n.	spa	irr-pi	10	no	5–13	1–2.5	2–50	irr	0.07–0.06	no	0.14	0.14		0.08 × 0.06	not found	w		w	

Colony growth: bu, bushy; fla, planar growth, flabelliform; spa, sparse growth.

Branching type: irr-pi, irregularly pinnate; lb, laterally branched.

Polyp distribution: irr, arrangement mostly in irregular longitudinal rows; reg, arrangement mostly in regular longitudinal rows.

Colors: dark orange (do), orange (o), pink (p), red (r), yellow (y), white (w).

X: character present.

Blank space: character absent or not found.

## Taxonomy

### Class Anthozoa Ehrenberg, 1831
Subclass Octocorallia Haeckel, 1866
Order Alcyonacea Lamouroux, 1812
Family Gorgoniidae Lamouroux, 1812
Genus *Eugorgia* Verrill, 1868

#### Eugorgia beebei sp. n.

http://zoobank.org/8B75AC49-5089-4BEF-BE80-B76198B9D0E8

http://species-id.net/wiki/Eugorgia_beebei

Figs 1 –3

Eugorgia rubens var. *beebei* (species name suggested by E. Deichmann in a museum label, unpublished)

##### Material examined.

**Holotype.** CASIZ 75783, ethanol preserved, Los Frailes, Baja California sur, México, 52 m, coll. R. Adcock, 18 June 1979.

**Figure 1.**
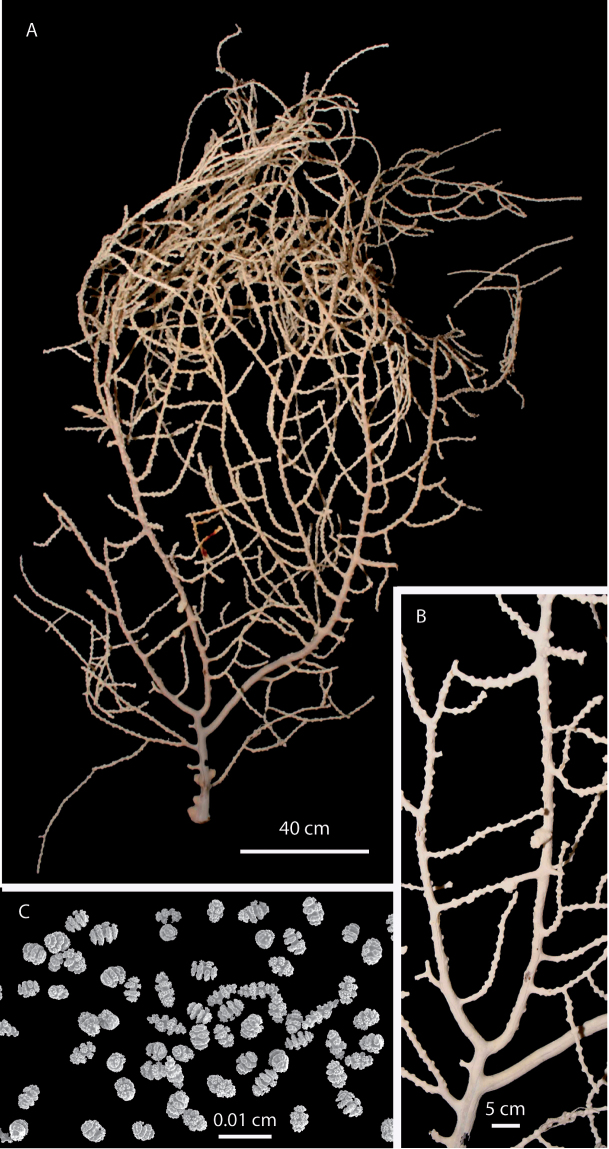
*Eugorgia beebei* (CASIZ 75783) holotype. **A** entire colony **B** detail of branches **C** SEM sclerites.

**Paratypes.** MCZ 36106, dry, Paita, Piura, Perú, no more data available. USNM 56879, ethanol preserved, El Alto, Piura, Perú, 1860-1815 m but depth data dubious (F. M. Bayer’s note on label: ‘specimen probably from previous shallow station’), *Anton Bruun* Cruise, 18B, Sta. 766, 4°10'S, 81°27'W, 9 September 1966.

**Figure 2.**
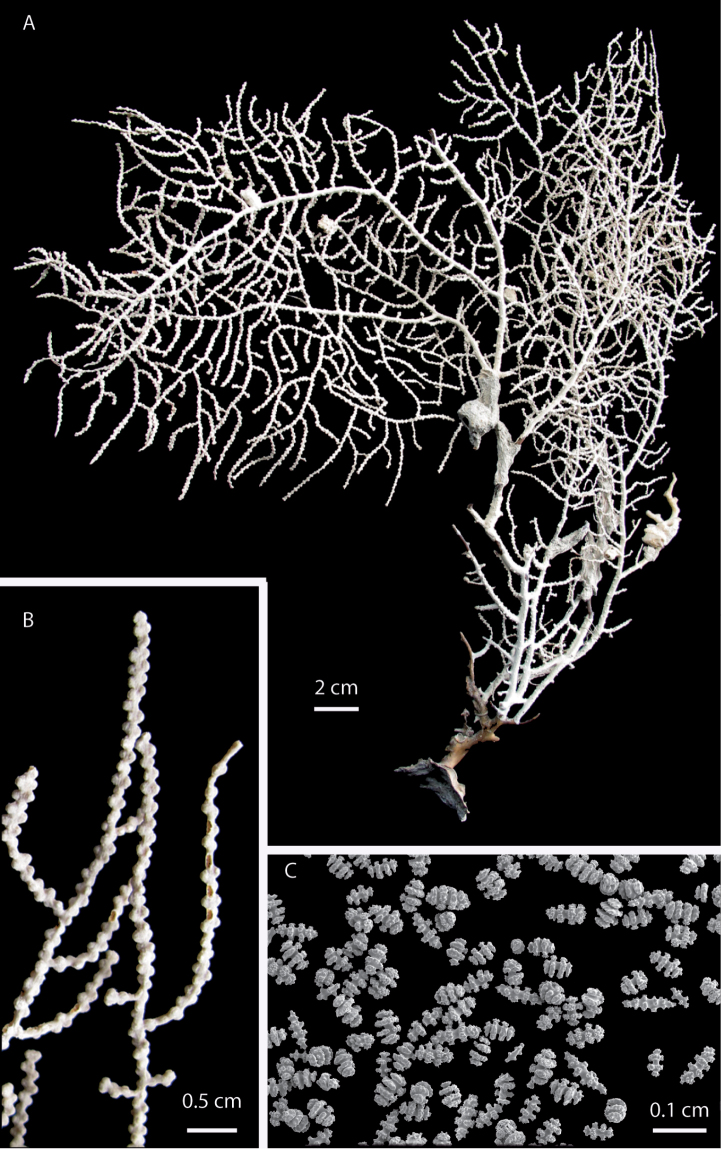
*Eugorgia beebei* (MCZ 36106) paratype. **A** entire colony **B** detail of branches; SEM sclerites.

##### Type locality.

Baja California sur, México.

##### Diagnosis.

Ascending colony sparse growing, branching irregularly pinnate, and multiplanar, subdividing up to 11 times, some pseudo-anastomosis present. Prominent polyp-mounds up to 0.70 mm tall, dome-shaped, arranged irregularly, and closely placed on branchlets, and very distant on thick branches. Colony and sclerites white. Spindles and disc-spindles up to 0.14 mm in length, double discs up to 0.07 mm long, and 0.05 mm wide. Anthocodial rods absent.

##### Description.

**Figure 3.**
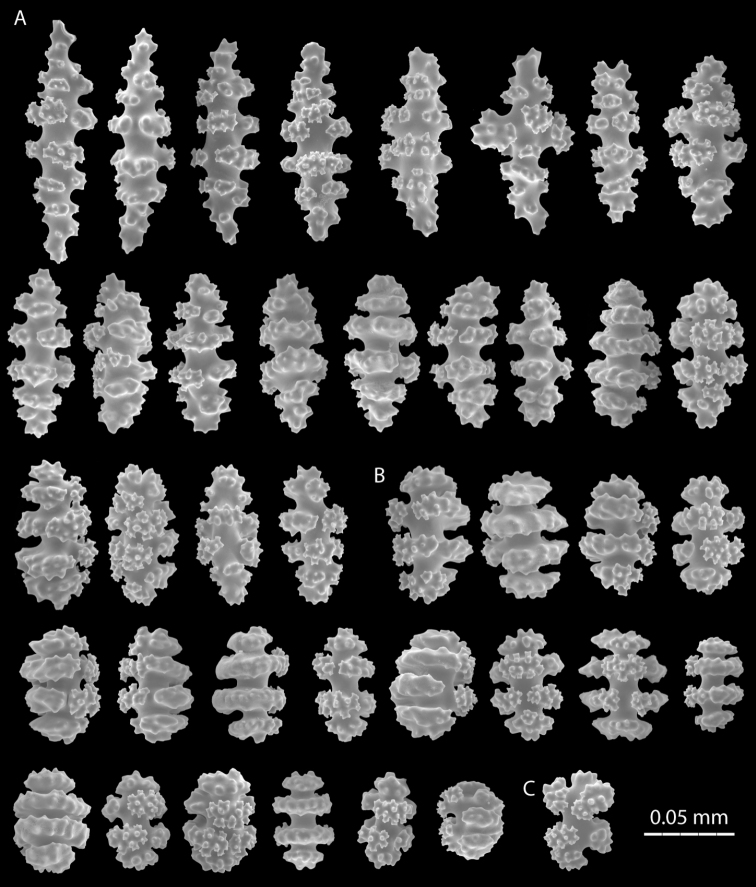
*Eugorgia beebei* (CASIZ 75783) holotype, SEM coenenchymal sclerites. **A** spindles and disc-spindles **B** double discs **C** cross.

Holotype 24 cm tall, and 20 cm wide, ascending, sparse growing, (Fig. 1A). Branching irregularly pinnate, and multiplanar, several pseudo-anastomosis occurs in branchlets and branches (Fig. 1A–B). Main stem 4 mm diameter at base, slightly compressed, and short, about 80 mm long arising from a fragment of holdfast, 0.6 mm diameter. Main stem gives off several branches and stumps. The three main branches, 3.0–4.0 mm in diameter, emerging at angles of 45–90°and producing secondary branches subdividing and giving off thin branchlets, up to 2.5 mm diameter, including polyp-mounds. Branchlets irregularly arranged, separated 5–16 mm, and giving off 2 or 3 lateral, secondary branchlets, of same thickness and arrangement. Colony branching up to 11 times. Unbranched terminal twigs blunt, and reaching up to 50 mm long (Figs 1A–B). Polyp-mounds prominent, up to 0.7 mm height and 1 mm in diameter, dome-shaped, with slit-like apertures, arranged irregularly, close together along the branchlets, and very distantly distributed or absent along the thick branches (Fig. 1B). Holdfast devoid of polyps. Colony white (Fig. 1A–B). Sclerites of coenenchyme white, mostly double discs (Fig. 1C). Spindles and disc-spindles, up to 0.14 mm long and 0.04 mm wide, with 4 or 5 whorls of warty tubercles, the ends mostly blunt (Fig. 3A). Double discs up to 0.07 mm long, and 0.05 mm wide (Fig. 3B). Crosses about 0.08x0.06 mm, scarce on samples (Fig. 3C). No anthocodial sclerites present in the samples.

##### Variability.

Paratype MCZ 36106 reaches up to 34 cm tall, and 31 cm wide, the main stem 0.7 mm diameter, slightly compressed, and short, about 1.0 cm long arising from an oval holdfast 3.2 cm diameter, and 0.2 cm thick (Fig. 2A–B). Sclerites as in the holotype (Fig. 2C). The other examined specimens are smaller, but very consistent in all aspects with the holotype.

##### Discussion.

The morphology of the colony, i.e., irregular-pinnate branching and prominent polyps, immediately segregates the new species from the *ampla*-group, and suggest a similarity with *daniana*-, *rubens*- and *siedenburgae*-groups. *Eugorgia beebei* and *Eugorgia siedenburgae* differ from the species in the *daniana*-group, including *Eugorgia mutabilis* sp. n. (described below), firstly, in the colony growth, which is sparse and ascending in *Eugorgia beebei* sp. n. but bushy and profuse in *Eugorgia siedenburgae*, not flabellate as it is in the *daniana*-group species. Secondly, it differs in the branching patterns because branchlets in the *daniana*-group form flat pinnate fronds with pinnae projecting in the same plane. That is not the case in *Eugorgia beebei* and *Eugorgia siedenburgae* where the secondary branchlets stick out in several, irregular planes.

*Eugorgia siedenburgae* and *Eugorgia rubens* form monospecific groups, they differ especially in the colony growth. The *rubens*-group have pink, sparse and laterally branched colonies, and the *siedenburgae*-group, have bushy, bicolored colonies (Breedy and Guzman 2013).

*Eugorgia beebei* and *Eugorgia siedenburgae* are very similar in sclerite content (Table 1), but they are different especially in the growth form and in the color. The conspicuous bushy colony immediately distinguished it from *Eugorgia beebei*; additionally, *Eugorgia beebei* has thicker branches and branchlets than *Eugorgia siedenburgae*; the polyp mounds are pointed and higher in the latter, and are more rounded in *Eugorgia beebei*. Branchlets in *Eugorgia beebei* are longer than in *Eugorgia siedenburgae* (Table 1).

##### Remarks.

We found the paratype in the MCZ (36106), labelled in Elisabeth Deichmann’s handwriting (Ardis Johnston, pers. comm.) as a variety of *Eugorgia rubens*, however, she certainly had not published anything on this genus, thus the variety or the species was never established. The specimen was part of an MCZ public exhibition, the only data we have are the locality. It is probable that this specimen examined by Deichmann came from the Zaca expedition of 1937 and 1938. We do not consider *Eugorgia beebei* as a variety of *Eugorgia rubens* because they differ in the traits that have been shown to be informative to separate species in the genus: color, branching pattern, colony growth and sclerite content (see Table 1), as mentioned above.

##### Etymology.

This new species of *Eugorgia* is named for explorer/naturalist William Beebe (1877–1962) who studied the marine fauna at numerous locations along the west coast of Central America from Mexico to Columbia during the Templeton Crocker *Zaca* expedition between 1937 and 1938. Beebe subsequently wrote the book, *Book of Bays*, which chronicles the five month expedition (Gould 2004).

##### Distribution.

Presently known from Piura, Perú and Baja California, but it is very likely that it exist along all along the geographic range. The depth range is 50–60 m, it is possible the range could extends deeper, but not as deep as reported for paratype USNM 56879, which is probably a mistake, as was remarked by F. M. Bayer (former USNM curator).

#### Eugorgia mutabilis sp. n.

http://zoobank.org/B552B4F0-50F4-4E16-BC00-5A3CB2C565C5

http://species-id.net/wiki/Eugorgia_mutabilis

Figs 4 –6

##### Material examined.

**Holotype.** UCR 2297, ethanol preserved, Burbujas, between Los Potreros and Playa Arenitas, Puerto Jiménez, Golfo Dulce, Costa Rica, 11 m, O. Breedy, 9 May 2013. **Paratypes.** UCR 2298, same data as the holotype. UCR 2272, 2276, fragments, ethanol preserved, Burbujas, 12 m, C. Sánchez, May 2012. UCR 2299, fragment, ethanol preserved, Roca Matapalo, Cabo Matapalo, Golfo Dulce, 20 m, O. Breedy, 6 February 2009. STRI 408, dry, Isla Seca Grande, Gulf of Chiriquí, Panamá, 20 m, H. Guzman, 26 August 2002. STRI 444, dry, Isla Jicarita, Gulf of Chiriquí, 20 m, H. Guzman, 29 August 2002. STRI 511, dry, Isla Ladrones, Gulf of Chiriquí, 15 m, H. Guzman, 14 April 2003. STRI 1073, Santa Cruz, Coiba Island, Panamá, 15 m, H. Guzman, 27 April 2007. STRI 1076, Twin Peaks, Coiba Island, 15 m, H. Guzman, 27 April 2007. STRI 1168, fragment, ethanol preserved, Bajo Hacha, Coiba Island, 20 m, O. Breedy, 16 April 2009. STRI 1122, ethanol preserved, La Blanca, Oxaca, Mexico, 46 m, R. Abeytia, 29 August 2004.

**Figure 4.**
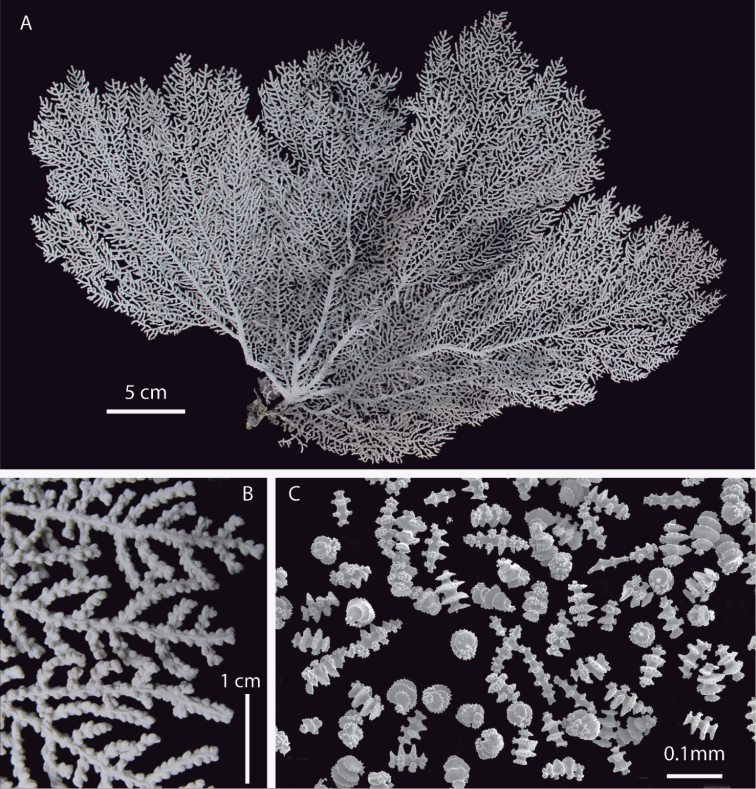
*Eugorgia mutabilis* (UCR 2297) holotype. **A** entire colony **B** detail of branches **C** SEM sclerites.

**Figure 5.**
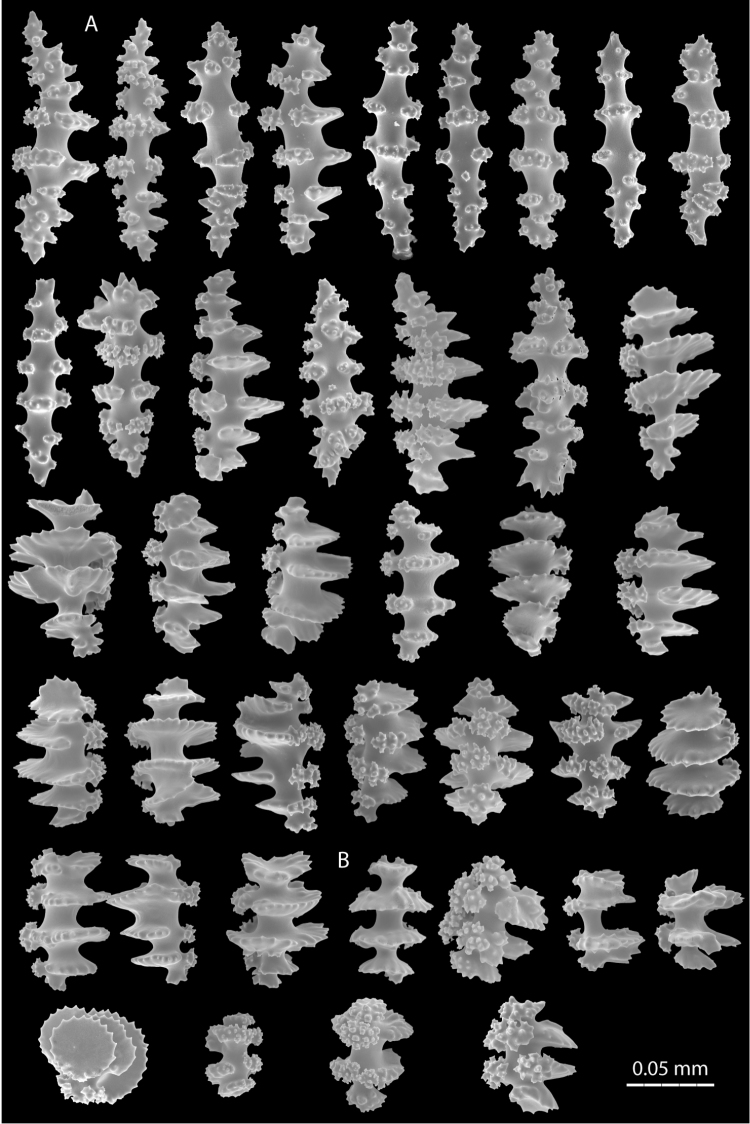
*Eugorgia mutabilis* (UCR 2297) holotype. SEM coenenchymal sclerites. **A, B** spindles and disc-spindles **C** double discs.

##### Type locality.

Puerto Jiménez, Golfo Dulce, Costa Rica, 11 m.

##### Diagnosis.

**Figure 6.**
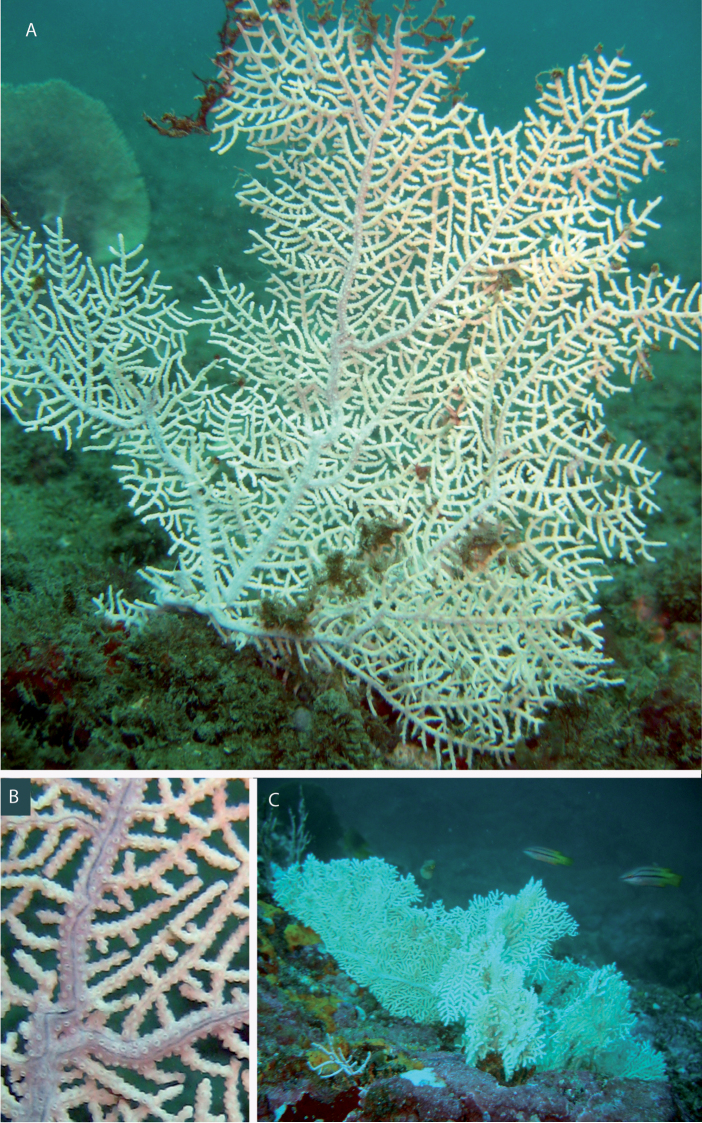
*Eugorgia mutabilis*, in situ colonies. **A, B** Burbujas, Puerto Jiménez, Golfo Dulce, Costa Rica, 12 m deep (photographs by C. Sánchez) **C** Montaña Rusa, Islas Contreras, Panamá, 32 m deep.

Broad, stout, flabellate colony, main branches sinuous, branching irregularly pinnate, subdividing 5–7 times, no anastomosis present. Prominent polyp-mounds closely spaced and irregularly distributed around branches and branchlets (Figs 4, 6A-C). Colony white, pale pink when alive (Fig. 6A–C), dark grayish when dry or ethanol preserved. Change in color after collection very conspicuous. Longitudinal grooves evident along branches and branchlets. Sclerites white. Spindles and disc-spindles up to 0.15 mm long, double discs mostly 0.05–0.08 mm long. Sclerite discs sharp, serrated and prominent. No anthocodial rods found.

##### Description.

Holotype 30 cm tall, and 47 cm wide; colony broad, flabellate, very flexible. Branching irregularly pinnate. Main stem 6 mm diameter, laterally flattened, and short, 14 mm long. Holdfast oval, 40 mm diameter without polyps. Main stem subdividing in 5 sinuous main branches. Main branches slightly flattened on plane of colony, 3–4 mm in diameter emerging at angles of about 45°, bifurcating and diverging producing five flat pinnate fronds of long pinnate branchlets (Fig. 4A–B). Pinnae short, up to 8 mm long, and 1.5–2 mm diameter including polyp-mounds, close together 1–3 mm apart. Branching up to 7 times. Unbranched terminal twigs blunt, and reach up to 8 mm long (Fig. 4A–B). Longitudinal grooves distinct along branches and pinnate branchlets, evident in living and preserved specimens. Polyps white (Fig. 4B). Polyp-mounds prominent, up to 0.7 mm height and 0.8 mm in diameter, arranged mostly in lateral rows along the branchlets and separated by the longitudinal grooves, and more sparsely and irregularly distributed along the thicker branches (Fig. 4B). Colony white to pale pink when alive, gray to dark grayish in ethanol/dry preservation (Fig. 4A). Change in color very conspicuous possibly by liberation of black pigments after collection. Sclerites of coenenchyme white. Sclerite discs conspicuous mostly sharp, serrated and prominent (Fig. 4C, 5A–B). Disc-spindles 0.08–0.12 mm long, and up to 0.06 wide with 4–5 whorls of discs (Fig. 5B); spindles and disc-spindles, longer and thinner up to 0.15 mm long and 0.05 mm wide, with 5–7 whorls of warty tubercles, the ends acute, blunt, or both (Fig. 5A). Double discs up to 0.08 mm long, and 0.05 mm wide with prominent discs (Fig. 5B), some almost complete (Fig. 5B). No crosses, capstans or anthocodial sclerites present in samples.

##### Variability.

The specimens present some variation in sclerite color, white sclerites being dominant, but some pale yellow hues could be observed in the samples. In all other aspects they agree with the holotype, including the change of color from bright white alive or recently collected to grayish when fixed. It is interesting that after collection, the specimens discharge a black pigment that turns the water black or the alcohol, and the colony becomes gray. The specimens from Mexico represent the deeper record; they have been observed down to 50 m, meaning that the range of depth extends from 40 to 50 m as it is for *Eugorgia rubens*, *Eugorgia siedenburgae*, and *Eugorgia beebei*, but the morphology of the colony and sclerites remain the same described for *Eugorgia mutabilis*. Current flow and depth are some of the environmental factors that could influence interspecific variability in octocorals (Fabricius and Alderslade 2001), but in this case no effect was observed. The morphology of the colony and sclerite content are persistent along the depth range.

##### Remarks.

The species was mentioned before as a variety of *Eugorgia daniana*: ‘a white variety has been observed in shoals in Costa Rica and Mexico occurring together with the red form’ (Breedy et al. 2009). However, after examination of many specimens from various localities and depths, we found enough differences with respect to *Eugorgia daniana*, and to the other species in the group, especially in the color and the conspicuous sclerites, to establish *Eugorgia mutablilis* as a new species.

##### Habitat.

The new species is found on rocky substrates, in general with other species of gorgonians, including *Eugorgia daniana*, but in some places, it is the only *Eugorgia* present (Fig. 6A–C). Other gorgonians found normally inhabiting the same localities are *Pacifigorgia irene*, *Pacifigorgia stenobrochis*, *Leptogorgia alba*, and *Carijoa riseii*, which were very abundant in the type locality. A variety of associated invertebrates were found on the holotype and paratype UCR 2298, including ophiuroids, *Ophiotrhix* sp., and crustaceans, shrimps, *Periclimenaeus* sp. and abundant crabs, *Orthochela* sp.

##### Etymology.

The specific epithet is from Latin, *mutablilis*, changeable, in allusion to the change in color after collecting.

##### Discussion.

*Eugorgia mutabilis* belongs to the *daniana*-group with a characteristic flabellate colony composed of flat pinnate fronds, and irregular pinnate-branching pattern, and prominent polyp mounds. The white color of the colony and sclerites of *Eugorgia mutabilis* separates it from the rest of the group. However, the new species is similar to *Eugorgia daniana* in some features, e.g. maximum number of branches, branchlet distance, polyp distribution (see Table 1), but the sclerite composition is very different. The dominant sclerites in *Eugorgia mutabilis* have very sharp crested discs that are very consistent in all specimens revised from Mexico, Costa Rica and Panama, and along the depth range. These type of sclerites are distinct also from the ones in *Eugorgia beebei*, and in *Eugorgia siedenburgae*.

##### Distribution.

Records from Costa Rica, México and Panamá suggest a wide distribution, at least from Mexico to Panamá, but this has to be further explored. The deepest record in Panama is 35 m, in Costa Rica 25 m, and in Mexico 50 m. Thus, the occurrence of this species from 11 to 50 m deep also suggests a large bathymetric range of distribution.

## Final remarks

There are not many morphological characters to differentiate species in octocorals, normally the combination of growth form, and size and color of colony and sclerites are the features used for identification. However, as it was acknowledged above, colony shape can vary within species in response to environmental conditions, light availability, wave exposure and currents (Fabricius and Alderslade 2001). Thus, species delimitation are sometimes difficult to draw. For this reason, the study of a reasonable amount of samples and habitats will be of aid to decide about morphological species and varieties, especially when accessibility to collection sites is possible. However, in some cases, only old museum specimens are available but identification and recognition for biodiversity accounts is imperative.

Molecular studies have been taken to understand boundaries between species and interspecific or intraspecific phylogenetic relationships; however, a complete molecular phylogeny has not been achieved due to the lack of molecular markers with adequate resolution to distinguish species (or sometimes genera) (Cairns and Bayer 2005, McFadden et al. 2006, McFadden et al. 2010, Williams 2013, Wirshing et al. 2005).

Morphological phylogenetic studies in *Eugorgia* (Breedy et al. 2009) have shown that color of the colonies and sclerites, similar colony morphology, and ecological habits are significant characteristics to separate clades. These characters alone are not informative enough. The combination of the characters used to separate the new species that are analyzed here, showed consistency when compared with other related taxa in the genus.

Four groups of *Eugorgia* have been proposed for the eastern Pacific, four species in the *daniana*-group, one in the *rubens*-group, onein the *siedenburgae*-group, and eight in the *ampla*-group.

Although, *Eugorgia beebei* sp. n. was first mentioned as a variety of *Eugorgia rubens*, we have demonstrated that it is a different species that does not even fit in the *rubens*-group, or in the other related groups (*daniana*-, *siedenburgae*-). Thus, a new group is here proposed, the *beebei*-group characterised by white colonies and sclerites, and with ascending, sparse colony growth.

The diagnostic characters of the *daniana*-group are herein modified adding the white color for colony and sclerites, to include *Eugorgia mutabilis* sp. n. in the group. It is important to mention that occurrence of complete double discs sclerites in these species-groups is scant, and the closest example to this type are the ones in the new species. Actually, after examined many specimens especially, in the *daniana*-group, the occurrence of neat complete double discs is not frequent. It seems a more common character of the *ampla*-group. The recently described *Eugorgia ahorcadensis* Soler-Hurtado & López-González, 2012 from Ecuador should be placed in the latter group. However, there is not enough evidence to separate this species from *Eugorgia nobilis* Verrill, 1868, from which it represents a morphological variety, basically with longer branches and darker sclerites. Therefore, herein we synonymize it with *Eugorgia nobilis*.

Presently, a total of 15 valid species are recorded for the eastern Pacific but this number should increase when more geographic areas and bathymetric ranges are explored. This research is a contribution to the knowledge of the eastern Pacific octocoral biodiversity.

## Supplementary Material

XML Treatment for Eugorgia beebei

XML Treatment for Eugorgia mutabilis
